# Effects of dietary chitin supplementation on body weight, haematology and serum biochemistry, fecal scores and gut microbiota of captive pangolins

**DOI:** 10.1186/s42523-026-00576-3

**Published:** 2026-04-24

**Authors:** Xinmei Wang, Chungang Xie, Rongquan Zheng, Shanjian Zheng, Yanni Wang, Jia Xu

**Affiliations:** 1 College of Life Sciences, Zhejiang Normal University, Jinhua, 321004 China; 2 College of Ecology and Agriculture, Sichuan Minzu College, Kangding, 626001 China; 3 Wildlife Protection and Management Station, Jinhua Municipal Bureau of Planning and Natural Resources, Jinhua, 321052 China; 4 Key Lab of Wildlife Biotechnology, Conservation and Utilization of Zhejiang Province, Zhejiang Normal University, Jinhua, 321004 China; 5 Department of Veterinary Medicine, Faculty of Agriculture, Jinhua University of Vocational Technology, Jinhua, 321000 China

**Keywords:** Chitin, Diet, Ex-situ Malayan pangolins, Gut microbiota, Termites

## Abstract

Termites are a natural component of the diet of Malayan pangolins, whereas artificial diets used under captive conditions may differ substantially from wild prey in structure and composition, particularly in chitin content. This study evaluated the effects of dietary supplementation with termites or chitin in seven captive male Malayan pangolins exposed sequentially to three dietary conditions: artificial diet alone, artificial diet supplemented with termites, and artificial diet supplemented with chitin. Body weight, hematological and serum biochemical parameters, fecal scores, and gut microbiota composition were assessed. The results showed that the effects of termite and chitin supplementation were not consistent. Compared with the other dietary phases, chitin supplementation was associated with body weight reduction in some individuals and changes in several physiological parameters, including white blood cells, lymphocytes, mean corpuscular volume, eosinophils, and even significantly reduced in phosphorus, calcium, albumin, and cholesterol (*p* < 0.05). In the gut microbiota analysis, termite supplementation significantly increased Chao1 richness and observed OTUs (*p <* 0.05), while the overall microbiota composition differed between the termite- and chitin-supplemented phases. In addition, the chitin-supplemented phase showed a higher relative abundance of some taxa previously reported to be associated with potential pathogenicity. In conclusion, supplementation with termites and chitin produced different physiological and microbiota responses in captive Malayan pangolins. Under the conditions of this study, supplementation with 3% chitin was associated with less favorable outcomes than termite supplementation. Future studies should further evaluate dietary composition, including chitin and other nutrient components, to support the development of optimized diets for captive pangolins.

Pangolins are myrmecophagous mammals of the genus *Manis*, order Pholidota, class Manidae, mainly feeds on ants and termites, whose exoskeletons are rich in chitin, such as the *Coptotermes formosanus* and the *Odontotermes formosanus*, etc. This dietary specialization is associated with morphological and physiological adaptations for processing insect-based diets.

In captive conditions, however, pangolins are typically fed artificial diets that differ substantially from their natural prey in both composition and physical structure. These discrepancies have been associated with poor dietary adaptation, gastrointestinal disorders, and nutritional imbalances, which remain major challenges for ex situ conservation and husbandry. There are some cases where pangolins experience gastrointestinal disorders and perish from malnutrition, due to the artificial food supplied is inappropriate for their digestive systems [1, 2]. There have also been intestinal parasites and nutritional failures caused by prolonged starvation [3]. Food inadaptability causes stomach perforation illness to kill even pangolins that can eat artificial food on their own [4].

The gut microbiota acts as a core regulator regulating host nutrient metabolism, immune homeostasis, and overall health, and its community structure and functional profiles differ remarkably across animals with distinct feeding habits and digestive physiologies. In ruminants (dairy cattle), the rumen microbiota is dominated by Firmicutes and Bacteroidetes, and is enriched with lignocellulolytic bacteria including *Fibrobacter*, *Ruminococcus*, and *Prevotella*. These microbes efficiently convert cellulose into volatile fatty acids through cascading degradation pathways, thereby supplying the main energy source for the host [5]. Gut microbial colonization in dairy calves is also modulated by early-life nutrition [6]. For monogastric omnivores (pigs), although the gut microbiota is also dominated by Firmicutes and Bacteroidetes, its cellulolytic capacity is relatively weak and fermentation occurs mainly in the hindgut. Supplementation with cellulose or fibrous feed ingredients can improve digestive enzyme activities and regulate intestinal intestinal homeostasis [7, 8]. As specialized myrmecophagous mammals, pangolins harbor gut microbiota predominantly composed of Firmicutes and Proteobacteria, and present a low capacity for cellulose degradation [9, 10]. Chitin is chemically distinct from cellulose and represents the main structural component of insect exoskeletons. It has been suggested to play a functional role in digestion and gut microbial ecology in insectivorous and myrmecophagous species [11–13]. Previous studies have identified chitin-degrading microorganisms in the intestines of Malayan pangolins (*Manis javanica*), and comparative genomic analyses indicate that myrmecophagous mammals retain chitinase-related genes and harbor gut microbiota enriched in chitinolytic taxa [14, 15]. These findings suggest that chitin may represent an important dietary component in these species. However, termites provide not only chitin, but also protein, lipids, and micronutrients, and therefore represent a complex nutritional matrix [2, 16]. It remains unclear whether supplementation with purified chitin alone can reproduce the physiological or microbiological responses associated with termite consumption in captive pangolins.

In this study, we evaluated the effects of supplementing an artificial diet with termites or chitin in captive Malayan pangolins. We assessed body weight, hematological and biochemical parameters, fecal characteristics, and gut microbiota composition. We hypothesized that chitin supplementation would influence physiological responses and gut microbiota, but that its effects may differ from those of whole-termite supplementation.

## Materials and methods

### Experimental animals

Seven adult male Malayan pangolins under human care were included in this study and housed at the Pangolin Conservation and Breeding Center in Zhejiang Province, China. All individuals had been maintained in captivity for approximately three years and were considered clinically healthy based on routine veterinary assessments. Detailed information on the animals is provided in Table 1.

**Table 1:** The detail information of Malayan pangolins under human care

Animals ID	Time in captivity (year)	Physiological state	Weight (kg)
MJ10	three	Health	6.39
MJ13	three	Health	9.52
MJ18	three	Health	9.45
MJ27	three	Health	6.66
MJ25	three	Health	6.57
MJ22	three	Health	5.26
MJ11	three	Health	5.64

### Experimental design and diet

The pangolins were sequentially exposed to three dietary conditions: (1) M0: artificial diet (baseline diet routinely used at the facility), (2) M1: artificial diet supplemented with 5% termites; (3) M2: artificial diet supplemented with chitin [17, 18].

The artificial diet consisted of dried black ants (*Polyrhachis vicina*), dried mealworms (*Tenebrio molitor*), commercial cat food, milk powder, and egg yolk, and was provided once daily at approximately 16:30. The daily feeding amount was approximately 50 g/kg body weight. Termites were sourced from Guangzhou, China, and chitin was obtained from a commercial supplier (Huantai County Jinhu Shell Products Co., Ltd.).

Each dietary phase lasted 37 days, including a 7-day adaptation period followed by a 30-day experimental period [11, 19]. The same individuals were used across all treatments (within-subject sequential design), with washout periods (~ 40 days) intervals between dietary phases to reduce potential carryover effects.

During the initial adaptation period for the chitin-supplemented diet, pangolins showed reduced acceptance of the 5% inclusion level. Therefore, the chitin level was gradually reduced to 3%, at which point normal feeding resumed [20]. This concentration was maintained for the remainder of the M2 phase.

### Sample collection

Fecal and blood samples were collected during May, June, and September 2022 under the three dietary conditions. All pangolins had been maintained under long-term captive conditions and were routinely handled by trained personnel as part of daily husbandry and veterinary care. Therefore, animals were habituated to human presence and handling prior to sampling. And no abnormal behavioral responses (e.g., excessive agitation or resistance) were observed during the sampling process.

Fresh fecal samples were collected on day 35 of each dietary phase. Enclosures were monitored regularly, and freshly voided feces were collected immediately using sterile disposable tools. Approximately 1–3 g of the inner portion of each fecal sample was aseptically transferred into sterile sampling tubes and stored appropriately for further analysis [21]. Blood samples were collected on day 37 between 08:00 and 10:30 by trained veterinarians via the caudal vein using sterile vacuum tubes. No anesthesia or chemical restraint was used [22]. Standardized handling procedures were applied to minimize stress.

### Sample processing and analysis

#### Hematology and serum biochemistry

Blood samples were processed for routine hematological and serum biochemical analyses, including white blood cell count (WBC), red blood cell count (RBC), hemoglobin (HGB), hematocrit (HCT), mean corpuscular volume (MCV), phosphorus (PHOS), calcium (Ca), albumin (ALB), and cholesterol (CHOL).

### Microbiome analysis

Fecal DNA was extracted using a commercial DNA extraction kit (Magnetic Soil and Stool DNA Kit) according to the manufacturer’s instructions. Extracted DNA samples were stored at − 80 °C ultra-low temperature refrigerator prior to being sent to Novogene Co., Ltd. for sequencing. DNA quality was assessed by quantitative PCR and agarose gel electrophoresis. Sequencing libraries were prepared and sequenced on the Illumina NovaSeq 6000 platform.

The V3–V4 region of the bacterial 16S rRNA gene was amplified using primers 341 F and 806R. Raw sequencing data were processed using standard pipelines, including quality filtering (Trimmomatic v0.33), read assembly (FLASH v1.2.7), and chimera removal (UCHIME v8.1) [23–25]. Operational taxonomic units (OTUs) were clustered using USEARCH v10.0, and taxonomic classification was performed using the RDP classifier against the SILVA database [26–29].

Alpha diversity indices (Chao1, observed species, Shannon, Simpson, and Good’s coverage) were calculated using QIIME2. Beta diversity analysis was conducted through PCoA (Principal coordinate analysis). LEfSe (Linear Discriminant Analysis Effect Size) analysis was conducted with an LDA score threshold of 4.

### Body weight and fecal scoring

Body weight was recorded every 12 days throughout the study. This interval was the body weight measurement interval of the study facility, it was selected to reduce handling frequency and minimize stress in this stress-sensitive species.

Fecal consistency was assessed as a non-invasive indicator of digestive response to dietary treatments. Fecal samples were visually evaluated by the same trained veterinary professional using a standardized scoring system (Table 2) [30, 31].

**Table 2:** Guidelines for fecal scoring in pangolins. Score the feces based on the description of the fecal state [31]

Fecal score	Description
1.0	Hard and extremely dry–crumbles when pressure applied.
1.5	Hard and dry with a distinct shape.
2.0	Well-formed without leaving a mark when touched.
2.5	Well-formed with a moist surface and a mark left when touched.
3.0	Moist and beginning to lose form. Definite mark when picked up.
3.5	Very moist with less of a definite form. No longer broken into distinct pieces.
4.0	Most form is lost; poor consistency; difficult to pick up.
4.5	Diarrhea with minor areas of solid consistency/shape.
5.0	Watery diarrhoea. No areas of consistency or shape.

### Sample size

A total of 23 fecal samples and 23 blood samples were collected across the three dietary conditions. Eight samples per type were obtained during the M0 and M1 phases, and seven during the M2 phase due to the loss of one individual prior to sample collection in this phase.

### Statistical analysis

Initial data were processed in Excel 2016 and subsequently analyzed using one-way ANOVA in SPSS 22.0, with post hoc comparisons performed using Duncan’s test to identify significant differences among groups. Statistical significance was set at *p* < 0.05.

## Results

### Changes in body weight

Body weight responses to the three dietary treatments are presented in Table 3. Significant differences among dietary phases were observed in four individuals (MJ10, MJ18, MJ22, and MJ27; *p* < 0.05), whereas no significant changes were detected in the remaining animals.

In these four individuals, body weight tended to decrease during the M2 phase compared with M0 and M1. No consistent pattern of weight change was observed across all individuals.

**Table 3:** Changes in body weight of Malayan pangolins during feeding with three distinct diets (mean ± SD)

Animals ID	Body weight (Kg)		
	Artificial diet (M0)	Artificial diet+termites (M1)	Artificial diet+chitin (M2)
MJ10	6.27 ± 0.11^a^	5.81 ± 0.10^b^	5.35 ± 0.36^b^
MJ11	5.74 ± 0.09	5.70 ± 0.21	5.30 ± 0.33
MJ13	9.68 ± 0.11	9.76 ± 0.27	9.53 ± 0.33
MJ18	9.54 ± 0.18^a^	9.22 ± 0.23^ab^	8.74 ± 0.37^b^
MJ22	5.39 ± 0.10^a^	5.40 ± 0.12^ab^	5.19 ± 0.10^b^
MJ25	6.70 ± 0.13	6.49 ± 0.01	6.29 ± 0.33
MJ27	6.71 ± 0.04^a^	6.48 ± 0.11^ab^	6.01 ± 0.24^b^

^a^Different letters on the same line indicate significant differences among the data (*p* < 0.05)

### Hematological parameters

Hematological parameters across dietary treatments are summarized in Table 4. Among the measured indices, only mean corpuscular hemoglobin concentration (MCHC) differed significantly between M0 and M1 (*p* < 0.05). Other parameters, including WBC, lymphocyte count (LYM), MCV, and eosinophil count (EOS), did not show statistically significant differences among treatments. However, several parameters showed a decreasing trend across sequential dietary phases (M0→M1→M2), although these changes were not statistically significant. The reference intervals derived from the literatures [22, 32–34].

### Serum biochemical parameters

Serum biochemical results are shown in Table 5. Significant differences were observed in PHOS, calcium (Ca), ALB, and CHOL among dietary treatments (*p* < 0.05). Compared with M0 and M1, PHOS levels were significantly lower in M2. Similarly, Ca levels were significantly lower in M2 than in M0. ALB and CHOL values showed a decreasing trend across dietary phases, with significant differences detected between M0 and M1 or M2 (*p* < 0.05). Some values fell outside reported reference ranges. The reference intervals derived from the literatures [22, 32–34].

**Table 4:** Effects of different diets on the blood routine of Malayan pangolins (mean ± SD)

Index	Unit	Artificial diet (M0)	Artificial diet+termites (M1)	Artificial diet+chitin (M2)	Reference interval	Reference
		Mean ± SD	Mean ± SD	Mean ± SD		
WBC	K/uL	7.32 ± 2.25	7.21 ± 1.03	5.33 ± 2.64	3.50–13.20	Chin et al., [35]
LYM	K/uL	5.89 ± 1.86	5.24 ± 1.35	4.31 ± 2.49	0.23–2.35	Ahmad et al., [33]
MONO (Monocyte)	K/uL	0.42 ± 0.15	0.51 ± 0.14	0.30 ± 0.14	0.02–0.84	Ahmad et al., [33]
RBC	M/uL	6.92 ± 1.17	6.50 ± 0.69	8.14 ± 2.86	3.47–8.62	Chin et al., [35]
HGB	g/dL	14.53 ± 2.12	13.81 ± 1.33	17.07 ± 5.75	8.30–18.60	Rupak et al., [22]
HCT	%	43.44 ± 7.54	39.09 ± 3.60	48.81 ± 17.17	30.50–51.70	Chin et al., [35]
MCV	fL	62.80 ± 2.71	60.21 ± 2.21	59.91 ± 1.92	58.60–82.30	Chin et al., [35]
MCH (Mean corpuscular hemoglobin)	pg	21.10 ± 0.98	21.27 ± 0.83	21.10 ± 0.83	20.10–28.90	Chin et al., [35]
MCHC	g/dL	33.60 ± 1.35^a^	35.33 ± 0.75^b^	35.17 ± 1.23^ab^	31.30–38.60	Chin et al., [35]
PLT (Platelet)	K/uL	85.86 ± 26.84	105.86 ± 32.64	92.29 ± 41.07	58.00-541.00	Chin et al., [35]
MPV (Mean platelet volume)	fL	7.93 ± 0.60	8.36 ± 2.00	7.86 ± 0.85		
PCT (Plateletcrit)	%	0.07 ± 0.02	0.09 ± 0.03	0.07 ± 0.03		
RETIC (Reticulocyte)	K/uL	9.23 ± 2.45	9.67 ± 4.61	9.24 ± 2.28		
EOS	K/uL	0.96 ± 0.45	0.70 ± 0.38	0.52 ± 0.52	0.02–0.40	Ahmad et al., [33]

^a^Different letters on the same line indicate significant differences among the data (*p* < 0.05)

**Table 5:** Effects of different diets on the blood biochemistry of Malayan pangolins (mean ± SD)

Index	Unit	Artificial diet (M0)	Artificial diet+termites (M1)	Artificial diet+chitin (M2)	Reference interval	Reference
		Mean ± SD	Mean ± SD	Mean ± SD		
GLU (Glucose)	mg/dL	65.14 ± 23.92	45.00 ± 7.87	51.43 ± 21.68	34.00-130.00	Chin et al., [35]
CREA (Creatinine)	mg/dL	0.46 ± 0.17	0.44 ± 0.10	0.26 ± 0.16	0.10–0.90	Chin et al., [35]
PHOS	mg/dL	5.49 ± 0.74^a^	5.23 ± 0.66^a^	4.10 ± 0.74^b^	4.10–7.30	Ahmad et al., [33]
Ca	mg/dL	9.80 ± 0.74^a^	9.60 ± 0.94^ab^	8.64 ± 0.53^b^	8.20-14.26	Ahmad et al., [33]
TP (Total protein)	g/dL	8.21 ± 0.86	7.53 ± 0.82	7.01 ± 0.80	5.20–9.60	Ahmad et al., [33]
ALB	g/dL	3.14 ± 0.35^a^	2.67 ± 0.21^b^	2.56 ± 0.33^bc^	2.70–4.50	Ahmad et al., [33]
GLOB (Globulin)	g/dL	5.06 ± 0.58	4.84 ± 0.60	4.46 ± 0.66	1.10–6.60	Ahmad et al., [33]
ALT (Alanine aminotransferase)	U/L	62.71 ± 21.16	84.86 ± 48.98	254.43 ± 176.09	49.00–188.00	Chin et al., [35]
ALP (Alkaline Phosphatase)	U/L	182.86 ± 47.31	234.00 ± 88.92	552.43 ± 334.52	109.00–442.00	Ahmad et al., [33]
TBIL (Total Bilirubin)	mg/dL	0.39 ± 0.09	0.49 ± 0.20	0.47 ± 0.13	0.10–1.80	Chin et al., [35]
CHOL	mg/dL	124.29 ± 47.47^a^	92.00 ± 31.99^a^	29.86 ± 17.68^b^	107.00-426.00	Chin et al., [35]
LIPA (Lipase)	U/L	282.71 ± 286.62	154.14 ± 49.89	164.43 ± 74.61	25.00-86.50	Rupak et al., [22]
BUN (Blood urea nitrogen)	mg/dL	22.71 ± 5.56	22.14 ± 7.99	28.71 ± 19.97	20.00–47.50	Chin et al., [35]

^a^Different letters on the same line indicate significant differences among the data (*p* < 0.05)

### Changes in fecal scores

**Fig. 1:**
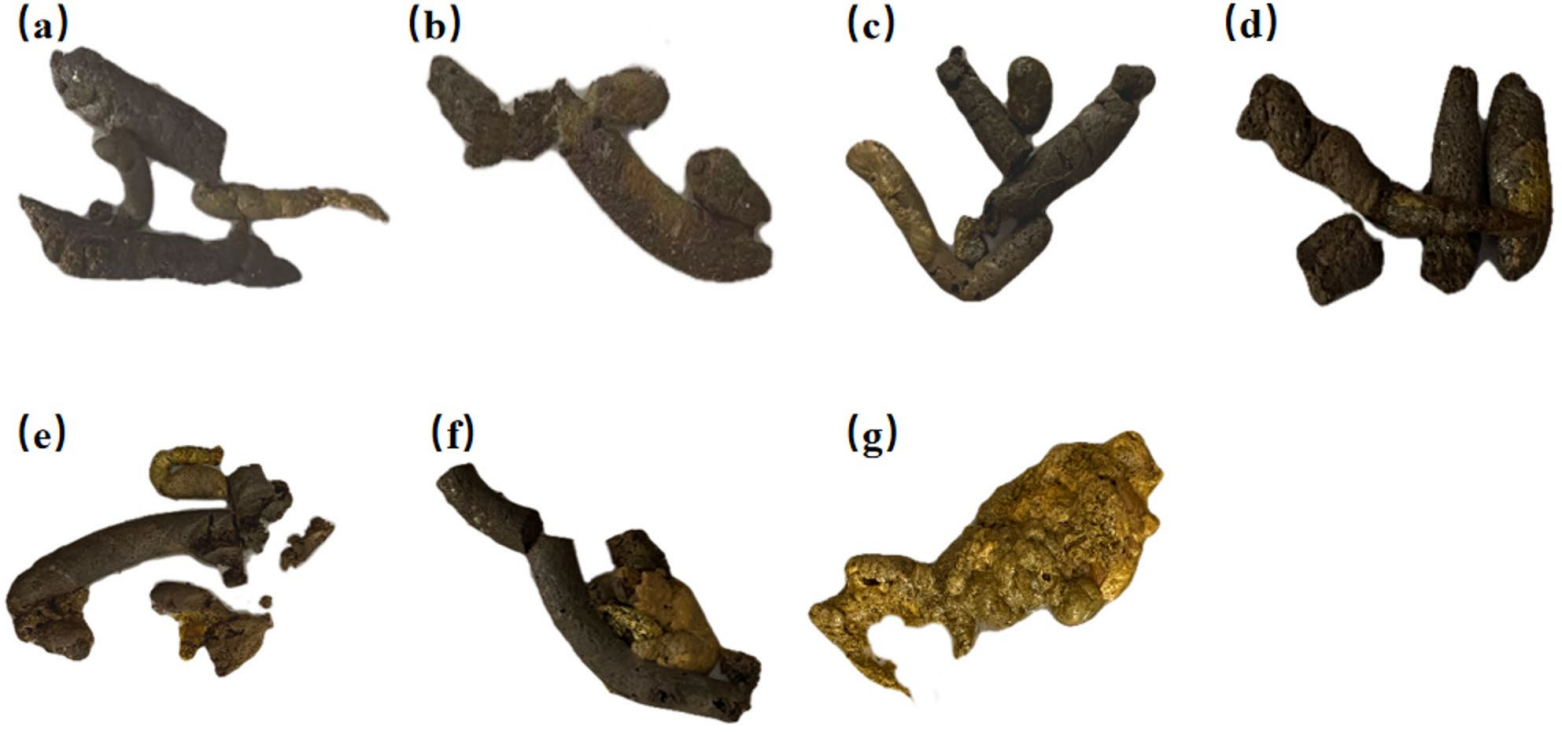
Legends for fecal scores of Malayan pangolins. These legends of pangolin feces were all taken directly during the experiment, and the fecal score was evaluated based on Clark’s research [31]. (**a**): fecal score: 1.5, (**b**): fecal score: 1.5, (**c**): fecal score: 2, (**d**): fecal score: 2, (**e**): fecal score: 2.5, (**f**): fecal score: 2.5, (**g**): fecal score: 4.5

During M0 and M1, fecal scores remained relatively stable (mean ± SD: 2.50 ± 0.00). During M2, fecal scores showed greater variability (1.95 ± 0.54 to 2.72 ± 0.75; Fig. 1a-b and c-g), with an overall shift toward softer fecal consistency.

### Changes in intestinal microbiota of pangolins under human care across different feeding phases

#### Diversity analysis of intestinal microbiota

**Fig. 2:**
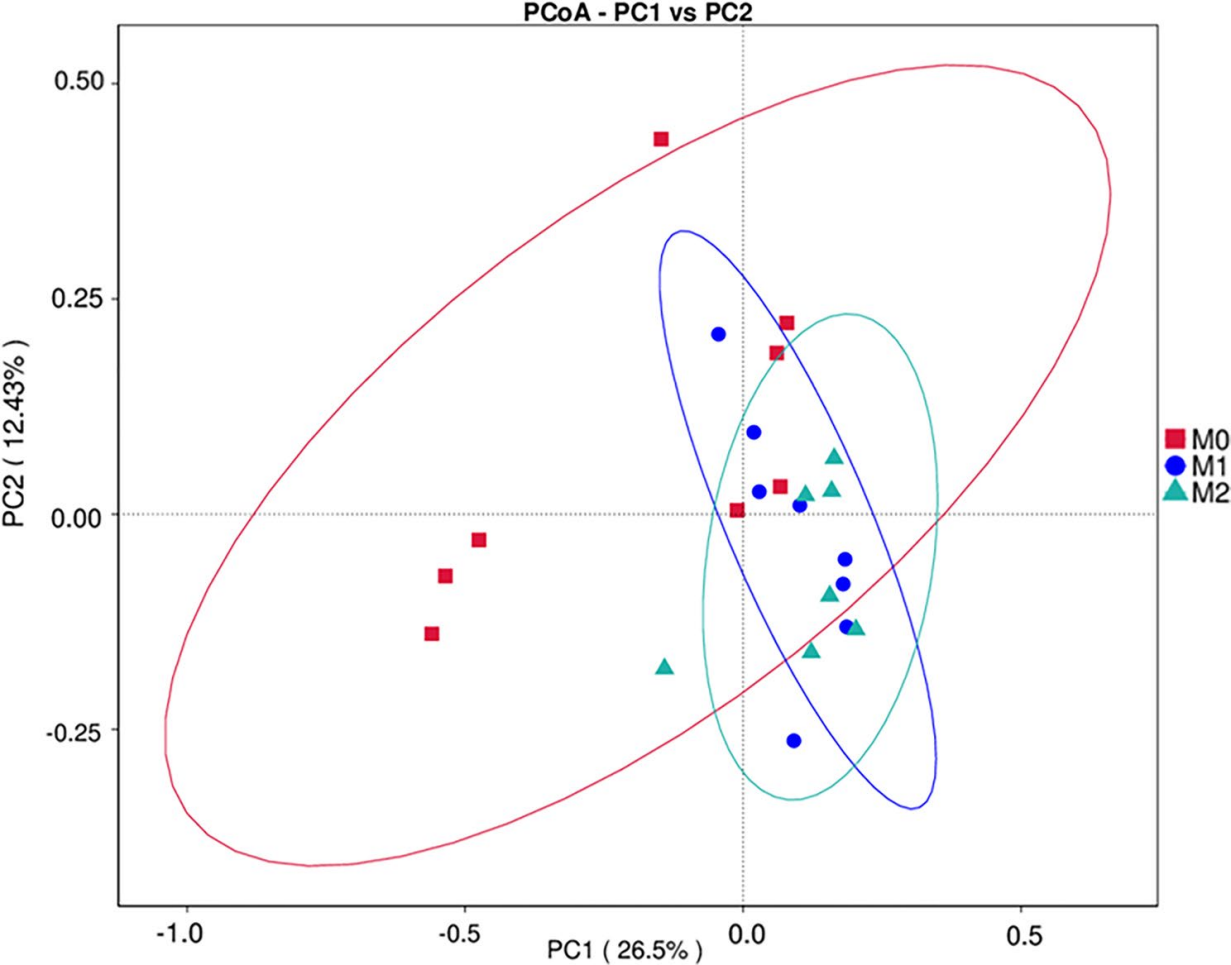
PCoA scatter plot presented the similarity in bacterial composition of Malayan pangolin across different dietary groups. Different colors represented different experimental groups respectively, red (M0): artificial diet, blue (M1): artificial diet+termites, and green (M2): artificial diet+chitin

Alpha diversity indices are shown in Table 6. Significant differences were observed in observed OTUs and Chao1 richness between M0 and M1 (*p* < 0.05), with higher values in M1. This indicated that the number of observable OTUs and the community abundance of intestinal microbiota were higher during the M1 compared to the M0. No significant differences were detected in Shannon or Simpson indices among dietary treatments. Beta diversity is used to analyze the temporal and spatial changes in species composition, reflecting whether there is difference in bacterial communities between groups. PCoA based on Unweighted unifrac distance is shown in Fig. 2. Samples from M1 and M2 clustered more closely compared with M0. Analysis of similarities (ANOSIM) indicated significant differences in microbial community composition between M0 and M1 (*R* = 0.254, *p* = 0.005) and between M0 and M2 (*R* = 0.254, *p* = 0.025).

**Table 6:** Alpha diversity indices of intestinal microbiota in Malayan pangolins across different dietary groups (mean ± SD)

Parameter	Artificial diet (M0)	Artificial diet+termites (M1)	Artificial diet+chitin (M2)
Good’s coverage	1.00 ± 0.00	1.00 ± 0.00	1.00 ± 0.00
observed_otus	333.86 ± 44.00^a^	517.14 ± 37.60^b^	411.57 ± 44.69^ab^
Chao1	334.92 ± 44.25^a^	518.67 ± 37.68^b^	414.70 ± 44.60^ab^
Shannon	4.99 ± 0.29	5.60 ± 0.17	4.92 ± 0.18
Simpson	0.89 ± 0.03	0.92 ± 0.01	0.91 ± 0.01

^a^Different letters on the same line indicate significant differences among the data (*p* < 0.05)

### Composition and relative abundance of core intestinal microbiota

**Fig. 3:**
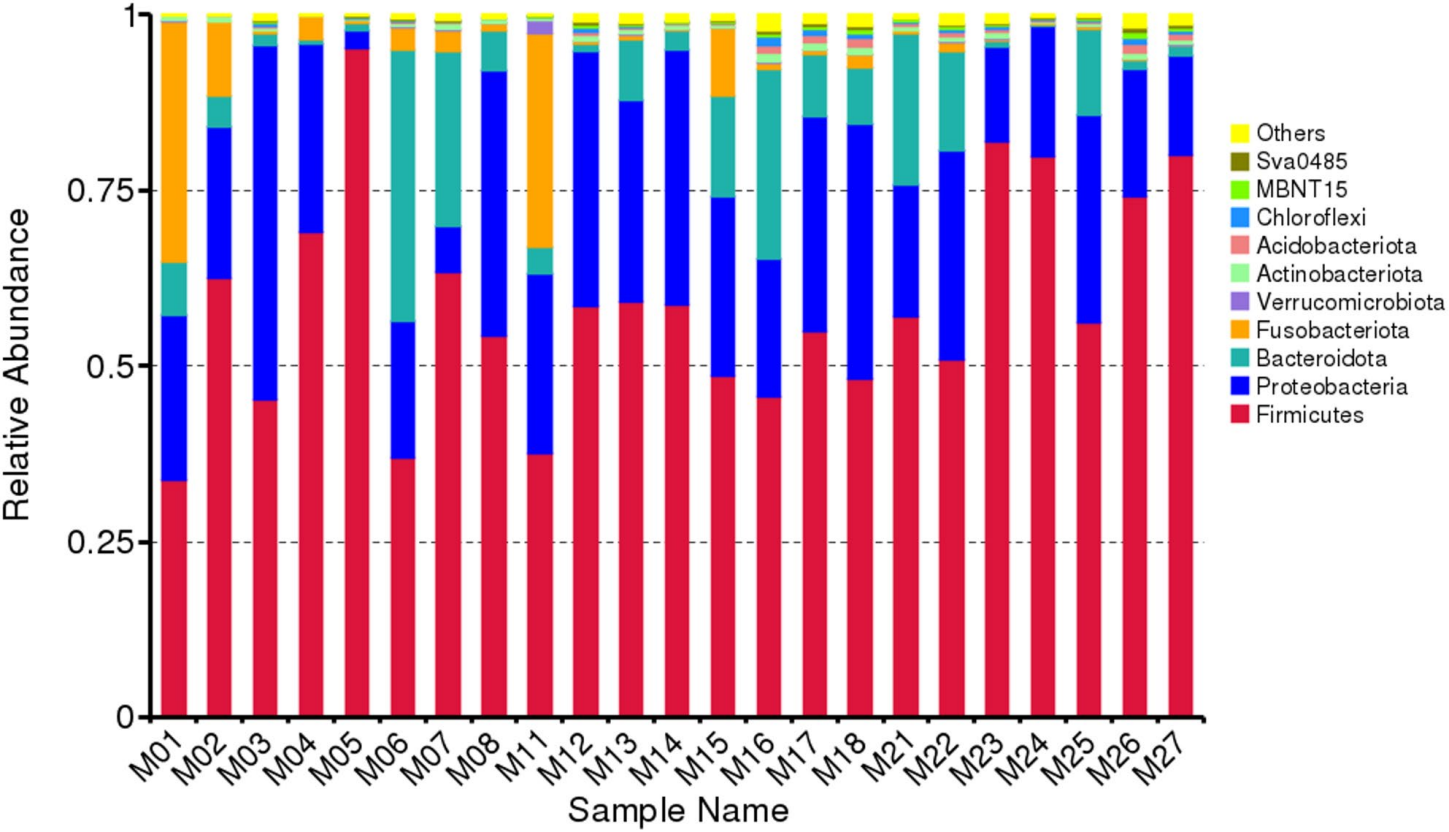
Dominant phyla in the intestinal microbiota of Malayan pangolins in different dietary groups

**Fig. 4:**
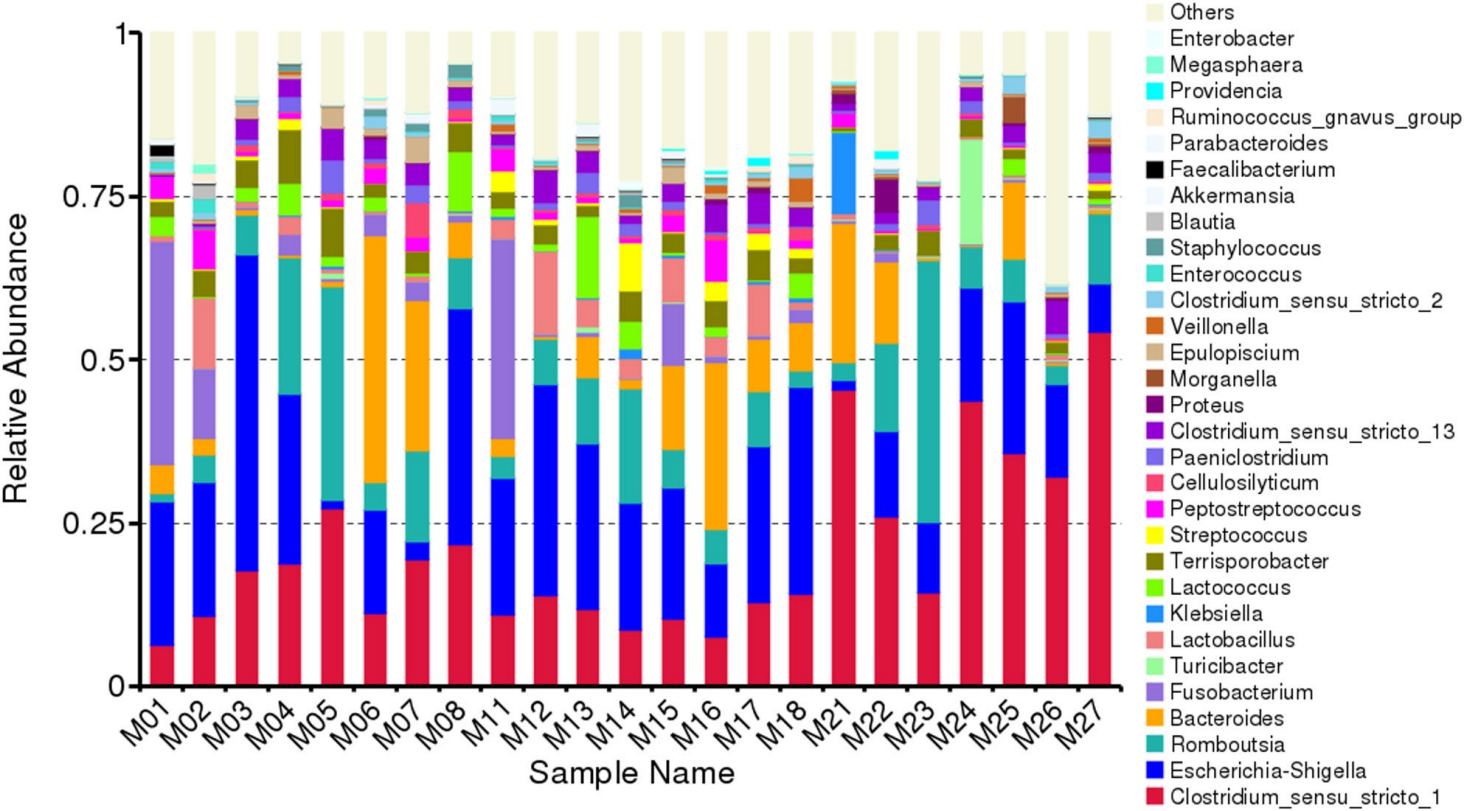
Dominant genus in the intestinal microbiota of Malayan pangolins in different dietary groups

At the phylum level (Fig. 3), Firmicutes, Proteobacteria, and Bacteroidetes were the dominant bacterial groups across all dietary treatments. Fusobacteriota ranked as the fourth most abundant phylum in M0 and M1, whereas Actinobacteriota ranked fourth in M2. At the genus level (Fig. 4), the dominant taxa differed among dietary treatments. In M0, the most abundant genera included *Escherichia-Shigella*, *Clostridium_sensu_stricto_1*, *Romboutsia*, *Bacteroides*, and *Fusobacterium*. In M1, *Escherichia-Shigella* remained the most abundant genus, followed by *Clostridium sensu stricto 1*, *Bacteroides*, *Romboutsia*, and *Fusobacterium*. In M2, *Clostridium sensu stricto 1* became the most abundant genus, followed by *Escherichia-Shigella*, *Romboutsia*, *Clostridium sensu stricto 13*, and *Turicibacter*.

### Species analysis of microbiota with significant differences

**Fig. 5:**
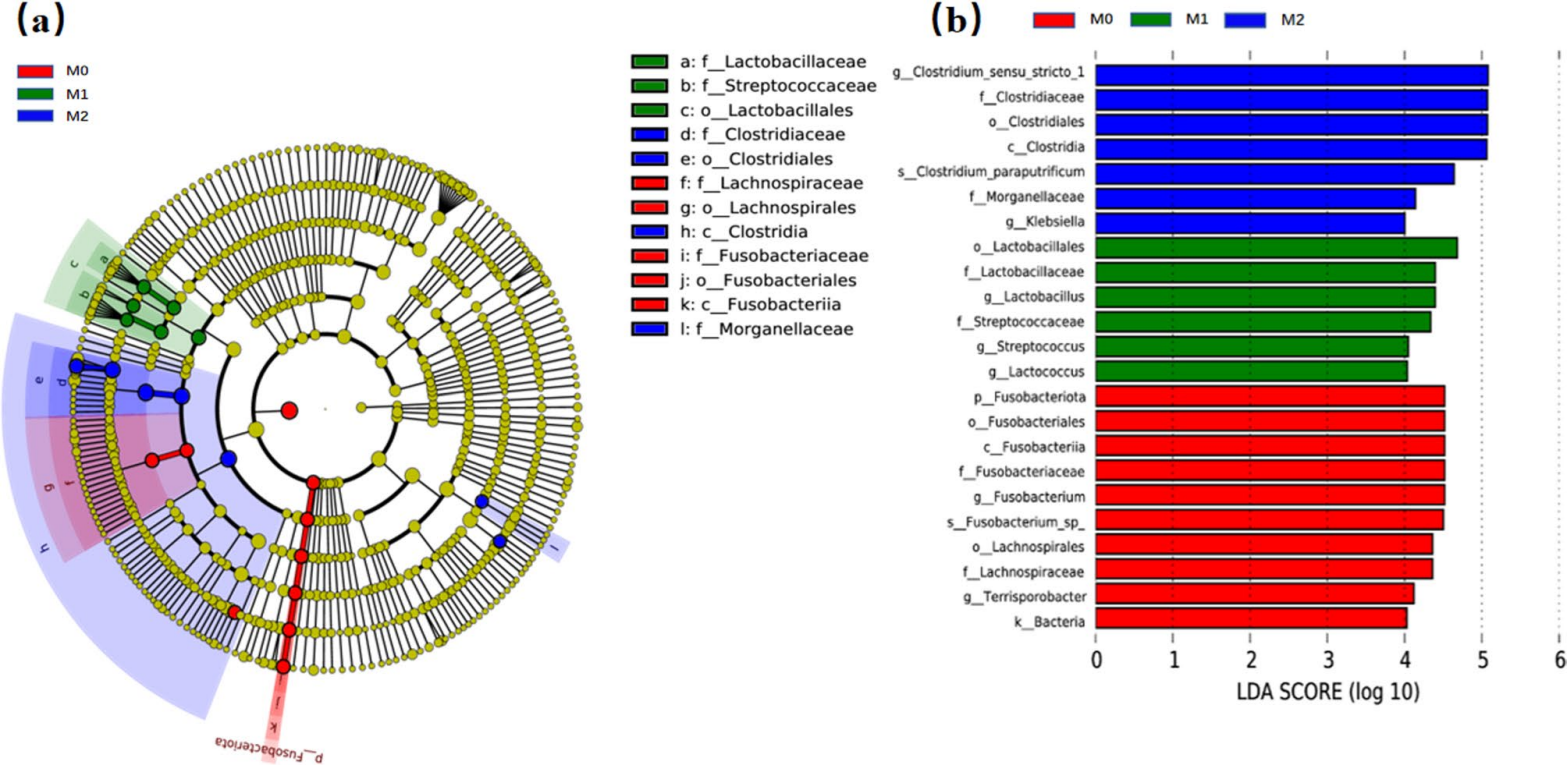
The results of LEfSe analysis (**a**) The cladogram diagram showed the microbial species with significant differences in the three groups. Red, green, and blue indicated different groups, with the species classification at the level of phylum, class, order, family, and genus shown from the inside to the outside. The red, green, and blue node in the phylogenetic tree represented microbial species that played an important role in the M0, M1, and M2 groups, respectively. Yellow nodes represented species with no significant difference. (**b**) Species with significant difference that have an LDA score greater than the estimated value; the default score was 4.0. The length of the histogram represents the LDA score; i.e., the degree of influence of species with significant difference between different groups

LEfSe analysis identified taxa that differed significantly among dietary treatments (Fig. 5). A total of 23 taxa were discriminative across groups. At the genus level, *Terrisporobacter* and *Fusobacterium* were enriched in M0, whereas *Lactococcus*, *Streptococcus*, and *Lactobacillus* were associated with M1. In M2, *Klebsiella* and *Clostridium sensu stricto_1* were identified as discriminative taxa.

## Discussion

The immune capacity of animals is influenced by numerous factors, with the quantity and quality of food being particularly critical, as both malnutrition and overnutrition can impair immune function in humans and animals [36]. In animals, nutritional status is frequently reflected through rapid weight changes, which subsequently affect immunity [37]. Body weight responses varied among individuals, with significant decreases observed in some pangolins during the chitin-supplemented phase. This variability likely reflected individual differences in dietary adaptation under captive conditions. Although weight loss was observed in some individuals, no consistent pattern was detected across all animals, suggesting that responses to dietary modification may be heterogeneous in captive pangolins. Overall, pangolin body weight demonstrated a progressive decline across all dietary phases (M0, M1, and M2), which might be related to the dietary composition and the longer intervals of body weight measurement.

Blood indices serve as valuable indicators for assessing animal physiological status. Research has established that RBC, HCT, HGB, MCV, MCHC, and other hematological parameters are influenced by physiological conditions, diet, exercise, and various environmental factors [38]. In the current study, pangolins exhibited a progressive decline in LYM, MCV, and EOS indices during sequential exposure to three dietary conditions. Previous studies indicate that decreased WBC and LYM levels may result from suppressed leukocyte and lymphocyte proliferation due to termite and chitin consumption [36]. The observed MCV decreased potentially correlates with enhanced pangolin activity during August and September, as increased activity expands erythrocyte surface area, improves gas exchange efficiency, and elevates circulatory velocity [39]. Parallel trends emerged in biochemical analyses, with ALB, CHOL, PHOS, and Ca demonstrating consistent declines across dietary phases. Notably, CHOL decreased precipitously to 29.86 ± 17.68 mg/dL during chitin supplementation, substantially deviating from reference ranges. While these changes may reflect alterations in nutritional intake or metabolism, the underlying mechanisms cannot be determined from the present study. In particular, reductions in albumin and cholesterol have been associated in other species with changes in nutritional status or metabolic function, but similar interpretations in pangolins require further investigation [40–45].

As a nocturnal species, the Malayan pangolin exhibits predominantly nocturnal defecation patterns. While prior research established an ideal fecal score range of 2-2.5 for pangolins, with soil and chitin supplementation improving digestive efficiency [11], our experimental results diverged from these findings. Fecal consistency remained relatively stable during the artificial diet and termite-supplemented phases but showed greater variability during chitin supplementation, including a tendency toward softer feces. As fecal scoring provides a general indicator of digestive response, these findings suggest that the chitin-supplemented diet may have altered gastrointestinal tolerance in some individuals. However, fecal scoring alone does not allow inference of specific digestive or microbiological mechanisms.

Gut microbiota analysis revealed differences in community structure and diversity among dietary treatments. Termite supplementation was associated with increased richness, whereas chitin supplementation resulted in shifts in both community composition and relative abundance of dominant taxa. ANOSIM analysis revealed significant differences among the M0 ~ M1, and M0 ~ M2 groups (*p* < 0.05), though the R-value of approximately 0.25 suggests that while the additives (termites or chitin) significantly altered microbial community structure, inter-group differences led to only some variations. The PCoA plot further showed that sample points for the M1 and M2 groups were more dispersed than those for M0, with greater within-group distances, indicating increased intra-group heterogeneity following additive supplementation. During the M0 and M1, *Lactobacillus* emerged as a dominant microbial species, potentially facilitating cellulose degradation, chitin digestion, and protein metabolism. However, chitin supplementation coincided with reduced *Lactobacillus* abundance, suggesting dietary composition and chitin source may influence microbial dynamics [46, 47]. The M2 exhibited unique dominance of *Turicibacter*, and was also the only experimental group where diarrhea was observed among the three dietary conditions. This association suggests chitin feeding may induce diarrheal symptoms in pangolins, and the addition of termites or chitin has increased the heterogeneity of the M1 and M2 bacterial community.

Several taxa differed in relative abundance among dietary groups, including *Lactobacillus*, *Turicibacter*, *Klebsiella*, and *Escherichia-Shigella*. These taxa have been reported in previous studies to be associated with a range of physiological or environmental conditions [15, 48–53]. However, the presence or increased abundance of these taxa does not imply pathogenicity or causal relationships with the observed physiological changes. Further studies incorporating functional and metabolomic analyses are required to clarify their roles.

Importantly, termites represent a complex nutritional matrix that includes not only chitin, but also protein, lipids, micronutrients, and other bioactive components. Historical analyses, such as Yang [54] study of three ant species (*P. vicina*,* Oecophylla smaragdinu* and *Macrotermes denticulatus*), revealed high protein content (49.92–61.52%) and rich mineral and vitamin profiles exceeding those of common foods like oils, meats, eggs, and fish. The termites used in this study (*O. formosanus*) contained 63.02% crude protein and 5.14% crude fat, values higher than those of common animals such as pork, grass carp, and silkworm moths, but lower than those of beef and mutton [55]. In contrast, purified chitin supplementation does not replicate this complexity. Therefore, the observed differences between termite and chitin treatments may reflect broader differences in dietary composition rather than the effects of chitin alone.

Several limitations of this study should be acknowledged. First, the sample size was limited, and all animals were exposed sequentially to the dietary treatments, which may introduce carryover effects. Second, the precise nutritional composition of the diets, including chitin content, was not analytically quantified. Third, microbiome analysis was based on amplicon sequencing and did not include functional or metabolomic validation. Future research should systematically evaluate different nutrient combinations to develop optimized diets for captive pangolins.

## Conclusions

In conclusion, termite and chitin supplementation produced distinct physiological and microbiota responses in captive Malayan pangolins. Under the conditions of this study, chitin supplementation at 3% was associated with less favorable outcomes compared with termite supplementation. Future studies should focus on optimizing dietary formulations by considering both structural components such as chitin and the broader nutritional composition of insect-based diets.

## Data Availability

The obtained data available for our study were submitted to NCBI Sequence Read Archive (SRA) under the study Accession Number: PRJNA1446698.
